# High Affinity Binding of *Escherichia coli* Cytotoxic Necrotizing Factor 1 (CNF1) to Lu/BCAM Adhesion Glycoprotein

**DOI:** 10.3390/toxins10010003

**Published:** 2017-12-21

**Authors:** Franziska Reppin, Sylvie Cochet, Wassim El Nemer, Günter Fritz, Gudula Schmidt

**Affiliations:** 1 Institute for Experimental and Clinical Pharmacology and Toxicology, Faculty of Medicine, Albert-Ludwigs-University of Freiburg, Albert-Street 25, 79104 Freiburg, Germany; reppin.franziska@gmail.com; 2 Biological Faculty, Albert-Ludwigs-University of Freiburg, Albert-Street 25, 79104 Freiburg, Germany; 3 Universite Sorbonne Paris Cite, Universite Paris Diderot, Inserm, INTS, Unite Biologie Integree du Globule Rouge, Laboratoire d’Excellence GR-Ex, 75013 Paris, France; sylvie.cochet@inserm.fr; 4 Department of Neuropathology, Albert-Ludwigs-University of Freiburg, Breisacher Strasse 64, 79106 Freiburg, Germany; guenter.fritz@uniklinik-freiburg.de

**Keywords:** Lu/BCAM, CNF, laminin, toxin, receptor, immunoglobulin-like domain, sickle cell disease

## Abstract

The protein toxin Cytotoxic Necrotizing Factor 1 (CNF1) is a major virulence factor of pathogenic *Escherichia coli* strains. It belongs to a family of single chain AB-toxins, which enter mammalian cells by receptor-mediated endocytosis. Recently, we identified the Lutheran (Lu) adhesion glycoprotein/basal cell adhesion molecule (BCAM) as a cellular receptor for CNF1. Here, we identified the Ig-like domain 2 of Lu/BCAM as main interaction site of the toxin by direct protein-protein interaction and competition studies. Using surface plasmon resonance, we showed a high affinity CNF-Lu/BCAM interaction with a KD of 2.8 nM. Furthermore, we performed small-angle X-ray scattering to define the molecular envelope of the Lu/BCAM-CNF1 complex, suggesting a 6:1 ratio of Lu/BCAM to CNF1 in the receptor-toxin complex. This study leads to a deeper understanding of the interaction between CNF1 and Lu/BCAM, and presents novel opportunities for the development of future anti-toxin strategies.

## 1. Introduction

Many Urinary tract infections (UTIs) are caused by Uropathogenic *Escherichia coli* (UPEC) strains [1], which produce the protein toxin Cytotoxic Necrotizing Factor 1 (CNF1). Like many other bacterial toxins, CNF1 modifies Rho proteins. Rho family GTPases are molecular switches, which are tightly controlled by three groups of proteins: GEFs (*guanine nucleotide exchange factors*), which activate Rho proteins by GDP/GTP-exchange; GAPs (*GTPase-activating proteins*), which stimulate GTP hydrolysis and thereby control the inactivation of Rho GTPases; and, GDIs (*guanine nucleotide dissociation inhibitors*), which predominantly bind the inactive form of Rho GTPases and block the nucleotide exchange [2]. In the active, GTP-bound state, Rho proteins interact with and activate several effectors. Best studied is the regulation of the actin cytoskeleton by Rho GTPases. Expression of active Cdc42 induces the formation of filopodia, active Rac stimulates membrane ruffling, and RhoA leads to stress fiber formation. CNF1 arrests Rho proteins in a constitutively activated state by deamidation of Gln61/63, which is required for GTP hydrolysis [3,4]. Consequently, cells that are treated with CNF1 show a strong induction of actin stress fibers, filopodia and membrane ruffles [5]. We and others showed that CNF1 destroys the barrier function of epithelia by opening tight junctions [6,7].

CNF1 is taken up into mammalian cells by receptor-mediated endocytosis [8]. It is released from the endosomes following acidification of the endosomal compartment and the cleavage of the toxin [9]. A hydrophobic part in the center of the molecule is required for membrane insertion and uptake of the toxins catalytic part into the cytosol [10]. Recently, we identified the Lutheran (Lu) adhesion glycoprotein/basal cell adhesion molecule (BCAM) as a cellular receptor for CNF1 [11]. Lu/BCAM is a transmembrane type-1 glycoprotein and a member of the immunoglobulin (Ig) superfamily containing five disulfide bonded extracellular Ig-like domains (for review see [12]). It is expressed in red blood cells and additionally shows a broad expression in epithelial and endothelial cells of several tissues [13,14,15]. Lu/BCAM binds to laminin α5, which is the major laminin α-chain in the basement membrane [16,17,18]. Mapping studies identified the first three domains of Lu/BCAM as critical for laminin binding [16,19]. Later, the Laminin binding site was mapped to domains 2 and 3 of Lu/BCAM by epitope mapping of an antibody competing with laminin-binding to Lu/BCAM [20]. By using X-ray crystallography, small-angle X-ray scattering and mutagenesis studies, the linker between domain 2 and 3 was identified as the binding site for Laminin [21].

In the present study, we investigated the binding properties of CNF1 and Lu/BCAM to get a deeper understanding of the toxin-receptor interaction. We studied the binding kinetics between CNF1 and Lu/BCAM and mapped their respective binding domains. We illustrate that the two proteins interact with high affinity. Moreover, we show that the toxin binds to the Ig-like domain 2 of Lu/BCAM. Finally, we created a molecular envelope of the CNF1-Lu/BCAM complex by performing small-angle X-ray scattering (SAXS) and suggest a ratio of 6:1 molecules Lu/BCAM to CNF1.

## 2. Results

In a previous study, we identified the Lutheran (Lu) adhesion glycoprotein/basal cell adhesion molecule (BCAM) as a cellular receptor for the bacterial toxin CNF1. Expression of the protein is necessary for toxin binding and lastly for the intoxication of the cells [11].

### 2.1. The C-Terminal Part of CNF1 Interacts with Lu/BCAM

**Figure 1:**
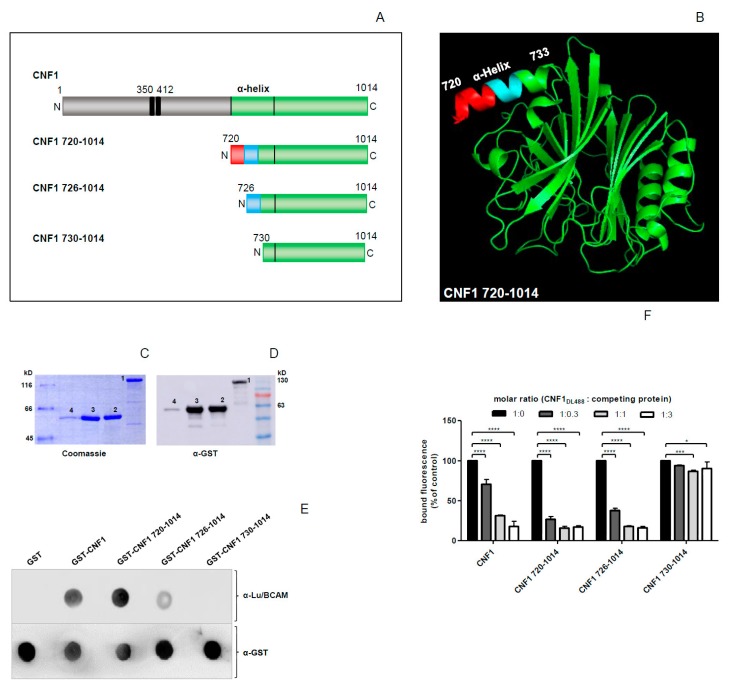
Cytotoxic Necrotizing Factor 1 (CNF1) 730–1014 shows no interaction, CNF1 726–1014 shows weaker interaction with rLu/BCAM in comparison to CNF1 and CNF1 720–1014. (**A**) Schematic representation of CNF1 and its truncated versions with the α-helix of interest highlighted; (**B**) crystal structure of the catalytic part of CNF1 from amino acid 720 to 1014, the α-helix of interest from amino acid 720 to 733 is highlighted; (**C**,**D**) enrichment of GST-CNF1 (1), GST-CNF1 720–1014 (2), GST-CNF1 726–1014 (3), and GST-CNF1 730–1014 (4) verified via SDS-PAGE following Coomassie staining (**C**) or an immunoblot using an anti-GST antibody (**D**); (**E**) dot blot binding analysis, different CNF proteins were dotted on a nitrocellulose membrane and incubated with rLu/BCAM for 30 min. After blocking the membrane with skimmed milk, detection of rLu/BCAM was performed with an anti-Lu/BCAM antibody; (**F**) flow cytometry competition studies, suspension of HeLa cells (3 × 10^5^ cells in 1 mL medium) were preincubated with different unlabeled N-terminal truncated CNF1 proteins, respectively. Then, cells were washed with PBS and incubated with DyLight488-labeled GST-CNF1 (as control cells were incubated with labeled CNF1 without preincubation and set to 100%). Following washing with PBS, cells were subjected to flow cytometry measurements. The bound fluorescence is shown in comparison to the control. Data shown represent three independent experiments + standard deviations. Statistical analyses were performed using two-way ANOVA. * *p* < 0.05; *** *p* < 0.001; **** *p* < 0.0001.

We have previously shown that the catalytic domain of CNF1 mediates Lu/BCAM binding [11]. This part of CNF1 (amino acids 720–1014) has been crystallized [22]. To narrow down the region of CNF1, which interacts with Lu/BCAM, we expressed and purified several truncated CNF proteins as GST-fusions (Figure 1). Precisely, we shortened the alpha helix depicted in Figure 1B, which seems to stick out from the global structure of the catalytic domain. To ensure the correct folding of GST-CNF1 fragments, we assessed the catalytic activity of the proteins by an *in vitro* Rho shift assay with recombinant RhoA. Deamidated RhoA (E63) runs at a higher molecular weight when compared to unmodified RhoA (Q63) (Figure S1A). All three of the truncated CNF proteins deamidated recombinant RhoA proving preserved catalytic activity of the CNF1 fragments and suggesting their correct folding (Figure S1A). We then analyzed direct protein-protein interaction of GST-CNF constructs and Lu/BCAM in dot blot assays. Therefore, we spotted the purified toxin fragments onto a nitrocellulose membrane and incubated it with recombinant Lu/BCAM (rLu/BCAM). Following washing steps, bound rLu/BCAM was analyzed by a specific antibody against Lu/BCAM and a peroxidase coupled secondary antibody (Figure 1E, top). As negative control, we used GST alone. To ensure equal protein loading, the membrane was probed additionally with an antibody against the GST tag (Figure 1E, bottom). As expected, rLu/BCAM interacted with GST-CNF1 and with GST-CNF1 (720–1014). Deletion of six amino acids from the alpha helix reduced CNF1-rLu/BCAM interaction (Figure 1E, top). Additional truncation of the alpha helix [GST-CNF1 (730–1014)] abrogated binding to rLu/BCAM, indicating that the alpha helix might be involved in toxin-receptor interaction. We further assessed the relevance of this mapping by performing competition studies on the cellular surface: HeLa cells were preincubated with unlabeled toxin fragments at different molar ratios, before adding DyLight-488-labeled full-length GST-CNF1. Following washing, binding of fluorescent CNF1 toxin to the cell surface was analyzed by flow cytometry. In accordance with the dot blot results, both GST-CNF1 (720–1014) and GST-CNF1 (726–1014) inhibited the binding of full-length CNF1 (Figure 1F). Interestingly, a molar ratio of 1:0.3 (labeled full-length CNF1: unlabeled GST-CNF1 (720–1014) or unlabeled GST-CNF1 (726–1014), respectively) was sufficient to significantly inhibit the binding of the full toxin. In contrast, a protein with an additional truncation of four amino acids [GST-CNF1 (730–1014)] only marginally influenced binding of the full toxin (Figure 1F). In contrast to the dot blot experiments with weak binding, GST-CNF1 (726–1014) blocked the binding of the full toxin in the FACS experiment. This may be due to the different temperatures being used in the experimental setups.

**Figure 2:**
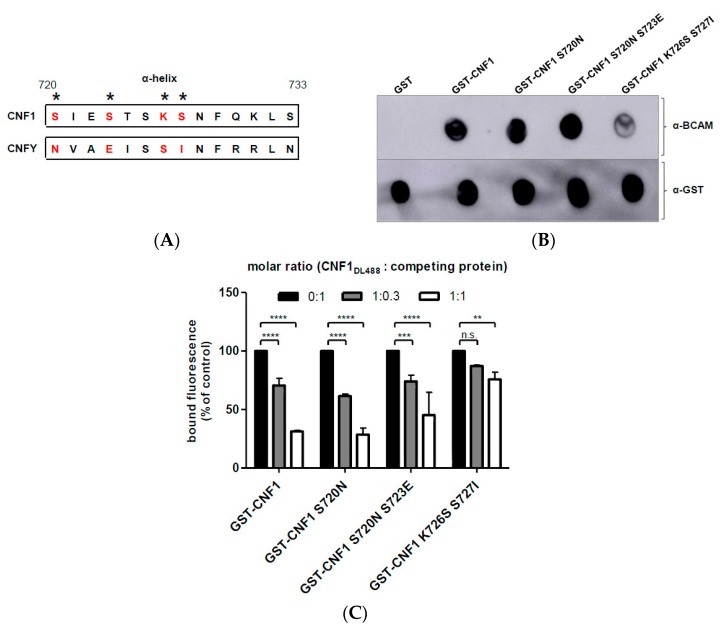
Analysis of the binding of CNF1 mutants to Lu/BCAM. (**A**) amino acid sequence of CNF1 α-helix of interest from amino acid 720 to 733 in comparison to CNFY, * indicate mutated amino acids; (**B**) dot blot binding studies, different CNF proteins were dotted on a nitrocellulose membrane and incubated with rLu/BCAM for 30 min. After blocking the membrane with skimmed milk, detection of rLu/BCAM was performed by an anti-Lu/BCAM antibody. As a loading control, the membrane was incubated with an anti-GST antibody; and, (**C**) flow cytometry competition studies, suspension of HeLa cells (3 × 10^5^ cells in 1 mL medium) were preincubated with several unlabeled CNF proteins in different molar ratios, respectively. Then, cells were washed with PBS and incubated with DyLight488-labeled GST-CNF1 (as control cells were incubated with labeled CNF1 without preincubation and set to 100%). Following washing with PBS, cells were subjected to flow cytometry measurements. Bound fluorescence is shown in comparison to the control. Data shown represent three independent experiments + standard deviations. Statistical analyses were performed using two-way ANOVA. ** *p* < 0.01; *** *p* < 0.001; **** *p* < 0.0001.

To finally map the interaction site within the alpha helix of CNF1, point mutations were introduced. We substituted single amino acids of CNF1 by corresponding residues of CNFY (Figure 2A). CNFY is a toxin that is produced by Yersinia pseudotuberculosis, sharing about 66% sequence identity with CNF1 [23]. CNFY does not bind to Lu/BCAM [11]. The structure of the CNF1 related toxin, can be modeled on the CNF1 crystal structure, suggesting high similarity. GST-CNF1 mutants were purified and determined as correctly folded by analyzing their activity, as described above. We then studied rLu/BCAM binding by dot blot analysis and by flow cytometry measurements as before. Only GST-CNF1 (K726S S727I) showed weaker binding to rLu/BCAM in comparison to GST-CNF1 (Figure 2B,C). The other mutants showed no effect. Because CNFY does not interact with Lu/BCAM, we then replaced the amino acids 720–733 of CNFY by the corresponding helix of CNF1 and vice versa and found that the resulting CNFY-chimera still did not bind to rLu/BCAM (Figure S1B–D), whereas the resulting CNF1-chimera bound to rLu/BCAM, regardless of the helix exchange. This shows that the alpha helix is not sufficient to mediate toxin-receptor interaction and that other parts of the catalytic domain add to the toxin-receptor interaction.

### 2.2. CNF1 Interacts with the Extracellular Ig-Like Domain 2 of Lu/BCAM

**Figure 3:**
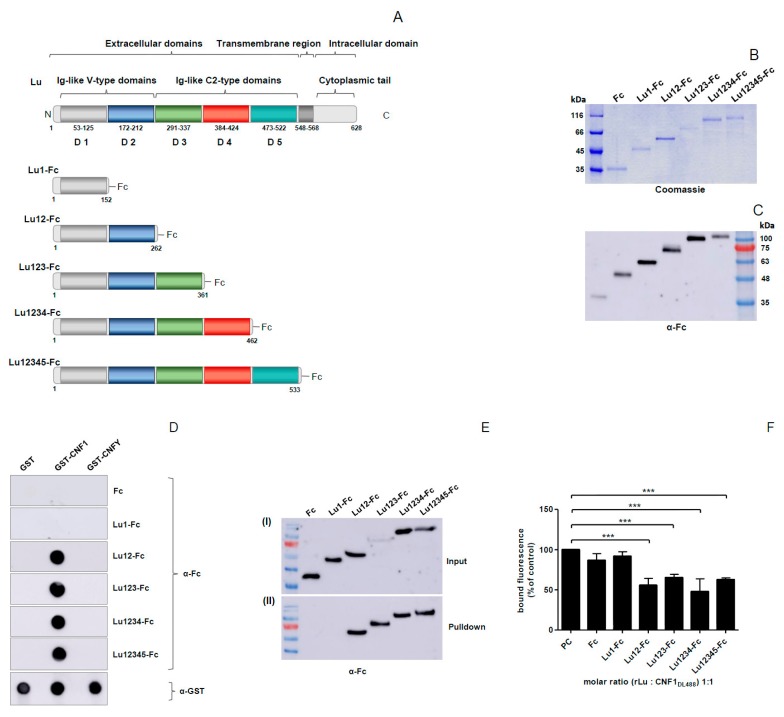
Domain 2 of Lu/BCAM is involved in binding to CNF1. (**A**) schematic representation of Lu with its extracellular, transmembrane and intracellular part; (**B**,**C**) purification of different Lu-Fc proteins and verification via SDS-PAGE (**B**) and western blotting using an anti-Fc antibody (**C**); (**D**) dot blot interaction studies, different CNF proteins were dotted on a nitrocellulose membrane, following blocking of the membrane with skimmed milk. After incubation with different Lu-Fc proteins, respectively, the Lu-Fc proteins were detected using an anti-Fc antibody. GST and GST-CNFY were used as negative controls; (**E**) GST pulldown assay with cell culture supernatants of Lu-Fc expressing COS7 cells. Cell culture supernatants of Lu-Fc expressing COS7 cells were incubated with beads-coupled GST-CNF1, respectively. After washing, the beads-coupled proteins were eluted with Laemmli sample buffer and separated by SDS-PAGE. Co-immunoprecipitation of Lu-Fc proteins was detected using an anti-Fc immunoblot (II). As Input, samples from the cell culture supernatants were taken before incubation with beads (I); (**F**) flow cytometry binding studies, DyLight488-labeled GST-CNF1 was pre-incubated with different Lu-Fc proteins respectively followed by an incubation with a suspension of HeLa cells (3 × 10^5^ cells in 1 mL medium). As positive control cells were incubated with DyLight488-labeled CNF1 without preincubation with Lu-Fc. After washing three times with PBS, cells were subjected to flow cytometry measurements. The bound fluorescence is shown in comparison to the positive control, which was set to 100%. Data shown represent three independent experiments + standard deviations. Statistical analyses were performed using one-way ANOVA. *** *p* < 0.001.

In a second set of experiments, we studied which part of Lu/BCAM is required for toxin binding. Therefore, the five extracellular Ig-like domains and domain combinations fused to a C-terminal Fc-tag (Figure 3A) were expressed in COS7 cells as secreted proteins. The different truncated Lu proteins were purified from the culture medium using Protein A sepharose beads and tested by SDS-PAGE, Coomassie blue staining (Figure 3B) and Western-blotting using an antibody against the Fc-tag (Figure 3C). For analysis of direct protein-protein interaction, equal amounts of GST, GST-CNF1, and GST-CNFY were spotted onto a nitrocellulose membrane that was later incubated with rLu/BCAM. GST and GST-CNFY served as negative controls. Bound Lu/BCAM was detected with an antibody against the Fc-tag. As shown in Figure 3D, the Fc-tag alone and domain 1 (Lu1-Fc) did not interact with any of the spotted proteins. In contrast, there was a specific interaction of all the proteins containing domains 1 and 2 (Lu12-Fc) with GST-CNF1 (Figure 3D). Addition of further domains did not influence binding to GST-CNF1 or allow binding to GST or to GST-CNFY. Unfortunately, the expression of domain 2 alone did not result in a stable protein. This is the reason why domain 2 without domain 1 could not be studied.

Additionally, we verified the interaction of the Lu/BCAM domains with GST-CNF1 in solution by pulldown assays. Therefore, beads-coupled GST-CNF1 was incubated with the culture medium of COS7 cells expressing different Lu-Fc proteins. Following washing and the elution steps, the presence of Lu-Fc proteins was analyzed by Western-blotting using an anti-Fc antibody. Consistent with the dot blot analysis, all of the fragments containing Lu/BCAM domain 2 were precipitated by beads coupled to GST-CNF1 (Figure 3E), confirming the interaction between this domain and CNF1.

Finally, competition between the toxin and the ligand (soluble Lu-Fc proteins) for receptor binding on HeLa cells was analyzed by FACS experiments using DyLight488-labeled GST-CNF1 and unlabeled Lu/BCAM domains. In this experiment, unlabeled Lu-Fc proteins and fluorescent toxin were incubated in equal molar ratios (1:1). Binding of DyLight-labeled GST-CNF1 to HeLa cells was assessed by determining the percentage of fluorescent cells. In line with the dot blot experiments, incubation of GST-CNF1 with Lu1-Fc did not inhibit the binding of the toxin to HeLa cells (Figure 3F). In contrast, Lu12-Fc and the other domain 2-containing Lu-Fc proteins reduced GST-CNF1 binding to the cells by about 50%. Taken together, these data indicate that domain 2 of Lu/BCAM is sufficient to mediate interaction with GST-CNF1, but it cannot be completely excluded that the C-terminal part of the linker region between domain 1 and 2 is involved in binding as all domain 2 containing proteins include this interdomain region.

### 2.3. High Affinity Binding of CNF1 to Lu/BCAM

**Figure 4:**
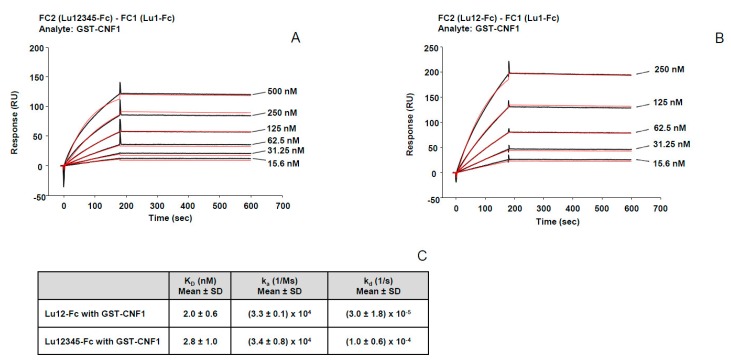
Lu12345 and Lu12 bind with similar affinity to CNF1. (**A**) Binding sensorgram for GST-CNF1 interaction with immobilized Lu12345-Fc. Lu1-Fc was immobilized on FC1, Lu12345-Fc on FC2. 6 different GST-CNF1 concentrations ranging from 15.6 nM, 31.25 nM. 62.5 nM, 125 nM, 250 nM up to 500 nM were injected. Shown is the sensorgram FC2-FC1; (**B**) binding sensorgram for GST-CNF1 interaction with immobilized Lu12-Fc. Lu1-Fc was immobilized on FC1 on a CM5 sensor chip, Lu12-Fc on FC2. As a ligand GST-CNF1 was injected at five different concentrations from 15.6 nM, 31.25 nM, 62.5 nM, 125 nM up to 250 nM. Shown is the sensorgram FC2-FC1. The red curves in (**A**,**B**) represent the fitted data estimated by the Biacore X100 Evaluation Software (Version 2.0, GE Healthcare, Little Chalfont, UK); (**C**) a table showing the affinity constant (K_D_), the association rate constant (k_a_) as well as the dissociation rate constant (k_d_) based on the kinetics calculated with the Biacore X100 calculation Software (Version 2.0, GE Healthcare, Little Chalfont, UK), *n* = 2; Mean ± SD. The presented results in (**A**,**B**) represent one of at least two independent replicates.

We further studied the affinity of CNF1 binding to Lu/BCAM by surface plasmon resonance using a Biacore. First, Lu12345-Fc was immobilized on a CM5 biochip and several concentrations of GST-CNF1 were injected. Increasing concentrations of GST-CNF1 yielded steeper binding curves, indicating a dose-response binding of GST-CNF1 to Lu12345-Fc (Figure 4A). Moreover, GST alone or GST-CNFY did not show any binding to Lu12345-Fc confirming the specificity of the interaction between Lu/BCAM and CNF1 (Figure S2A,B). We assessed the kinetics of GST-CNF1 binding to Lu12345-Fc, and found a K_D_ in the nanomolar scale (K_D_ = 2.8 × 10^−9^ M), indicating a high affinity between CNF1 and Lu/BCAM (Figure 4C). In addition, the curves showed a very weak dissociation between the two proteins (k_d_ = 1.0 × 10^−4^ s^−1^), suggesting a high stability of the complex that was formed. Next, in order to confirm our mapping results, similar experiments were performed with the Lu1-Fc and the Lu12-Fc constructs (Figure 4B). GST-CNF1 did bind to Lu1-Fc, but bound to Lu12-Fc in a dose-dependent manner with an affinity similar to its binding to Lu12345-Fc, suggesting that the CNF1 binding site is located in the second Ig-like domain of Lu/BCAM, without major additional binding sites in Lu/BCAM contributing to toxin-receptor affinity (Figure 4).

### 2.4. Modelling the CNF1–Lu/BCAM Complex

The high affinity of the two recombinant proteins allowed for us to purify the receptor-toxin complex by gel filtration experiments. Therefore, CNF1 (720–1014) and recombinant human Lu/BCAM with Fc-tag were preincubated at equimolar ratios for 20 min at 4 °C to form a stable complex. Following gel filtration, SDS-PAGE was performed to analyze the different fractions (Figure S3D). CNF1 (720–1014) has a molecular weight of 32 kDa, rLu/BCAM a size of approx. 100 kDa, according to the manufacturer’s instructions. By comparing the retention volume of rLu/BCAM (9.59 mL) (Figure S3B) and the complex (9.49 mL as mean of six experiments (Figure S3E,C) with a known protein standard and the corresponding exponential curve, the molecular weight of the different molecules were determined. rLu/BCAM in solution seems to form a hexamer with the size of approximately 571 kDa, together with CNF1 (720–1014) approx. 602 kDa. This leads to a 6 to 1 ratio of Lu/BCAM to CNF1.

**Figure 5:**
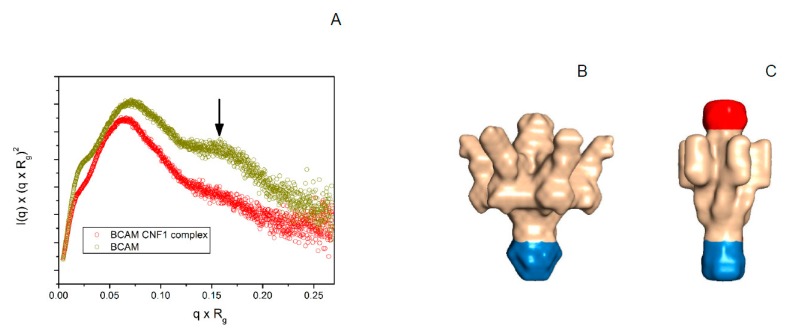
Small angle X-ray analysis of Lu/BCAM complex formation with CNF1. (**A**) Normalized Kratky plots of Lu/BCAM and Lu/BCAM-CNF1 complex. The Kratky plot of Lu/BCAM (dark yellow trace) shows that Lu/BCAM is an aspherical particle with large flexible areas as indicated by an arrow. In contrast, the normalized Kratky plot of the Lu/BCAM-CNF1 complex (red trace) reveals that the flexibility is largely reduced; (**B**) SAXS envelope reconstructed from the scattering curve with DAMMIF of Lu/BCAM assuming a six-fold symmetry as calculated from the size of the particle. The presumable Fc region of recombinant Lu/BCAM fusion protein is indicated in blue. The protruding arm-like structures correspond in size to the five Ig domains of the extracellular domain of Lu/BCAM, respectively; (**C**) The reconstructed envelope of Lu/BCAM-CNF1 complex shows additional density at the top end of the particle and indicates largely reduced flexibility of the arm-like structures, which assemble in the center of the molecule. This is strongly supported by the normalized Kratky plot depicted in (**A**).

The purified protein complex was used for crystallization studies. However, only small crystals that diffracted only to low resolution were obtained. To get an idea about the complex composition, SAXS experiments were performed with rLu/BCAM alone or with the purified rLu/BCAM-CNF1 complex. rLu/BCAM forms a covalent dimer due to the disulfide bridges between the Fc units. The SAXS data revealed for rLu/BCAM fusion protein a large molecular assembly with a volume of approximately 6.2 × 10^5^ Å^3^. This corresponds to a rLu/BCAM hexamer and is in agreement with the observation of Fc multimers in solution [24]; i.e., the assembly harbors six extracellular domains of Lu/BCAM. The normalized Kratky plot (Figure 5A) showed that the particle comprises a rigid and a rather flexible part. The rLu/BCAM-CNF1 complex forms a larger assembly, with a size of approximately 7.7 × 10^5^ Å^3^. The larger size of the particle indicates binding of 1–2 CNF1 molecules to the rLu/BCAM assembly and the Kratky plot clearly showed that the complex exhibits much lower flexibility than rLu/BCAM alone (Figure 5A).

With a content of six rLu/BCAM extracellular domains per particle, a six-fold symmetry was assumed in the DAMMIF analysis. The resulting molecular envelope for the rLu/BCAM assembly revealed a particle with a mushroom-like shape (Figure 5B). The cap of this particle represents most likely the flexible rLu/BCAM moieties that can adopt different conformations that are not resolved in the SAXS derived molecular envelope. The stem is quite compact and very likely represents the trimer of the Fc moieties.

In contrast, the rLu/BCAM-CNF1 complex revealed a more tailored and elongated particle, indicating the restricted flexibility of the rLu/BCAM moieties, whereas the stem region remains rather unchanged (Figure 5C). Strikingly, there is some extra mass observed when compared to the particle of rLu/BCAM alone. This noticeable portion most likely represents bound CNF1.

## 3. Discussion

We recently identified Lu/BCAM as the receptor for the Cytotoxic Necrotizing Factor 1 produced by uropathogenic *E. coli* and found that amino acids 720–1014, corresponding to the catalytic domain of CNF1, are important for this binding [11].

In the present study, we generated N-terminal truncations of the catalytic domain of CNF1 to identify the specific binding area to Lu/BCAM.

Results show that the deletion of amino acids 720–729 (CNF1 (730–1014)) completely abolished the binding to Lu/BCAM, whereas the removal of amino acids 720–725 (CNF1 (726–1014)) attenuated it.

These data show that amino acids 720–733 are involved in the interaction with Lu/BCAM. Interestingly, these residues are represented in an α-helix in the crystal structure of the catalytic domain of CNF1 [22].

Introducing point mutations into this helix identified one mutant (GST-CNF1 (K726S S727I)) that binds with a decreased affinity to Lu/BCAM. However, the insertion of the CNF1 α-helix (amino acids 720–733) into CNFY did not allow for binding of the related Yersinia toxin to Lu/BCAM. Moreover, the replacement of the amino acids 720–733 in CNF1 to the corresponding amino acids in CNFY did not attenuate or even abolish binding to Lu/BCAM. This shows that the amino acids 720–733 of CNF1 may be involved in the binding to Lu/BCAM, but are not sufficient to mediate the toxin-receptor interaction. Other parts of the catalytic domain are required to mediate high affinity binding to the receptor. Further studies are needed to clarify this matter.

Lu/BCAM is a type 1 transmembrane protein with five Ig-like extracellular domains (for review see [12]). All five Ig-like domains could interact with CNF1, either independently or together. By performing direct protein-protein interaction studies, flow cytometry binding assays and co-precipitation analyses, we showed that all rLu/BCAM proteins containing domain 2 bound to CNF1. The presence of domain 3, 4, and 5 neither increased nor decreased the binding. Surface plasmon resonance (SPR) measurements showed a K_D_ value of ~2.8 nM for the CNF1-Lu12345 interaction and a K_D_ value of ~2.0 nM for CNF1 binding to Lu containing only domain 1 and 2. This confirmed our data that the CNF1 binding site is located within domain 2. But, (additional) interaction within the last 20 amino acids of the linker region between domain 1 and 2 cannot be excluded, as all proteins containing domain 1 and 2 include this linker region. One interesting aspect of our studies is that besides the very high affinity between GST-CNF1 and rLu/BCAM, we also observed a very weak dissociation rate, which renders the complex that is formed by GST-CNF1 and rLu/BCAM very stable. The natural binding partner of Lu/BCAM, Laminin, shows a similar binding affinity constant of 10.8 nM [16]. The question occurs whether CNF1 and Laminin bind to the same part of Lu/BCAM. Mankelow et al. showed that Laminin binds to the flexible 2–3 interdomain region of Lu/BCAM [21]. A Lu fragment containing domain 1 and 2 (Lu12-Fc), without this flexible linker, showed no binding to laminin [16], whereas GST-CNF1 still bound to it in our experiments. Furthermore, flow cytometry assays revealed the interaction of GST-CNF1 to K562 cells expressing a Lu mutant D343R (see Figure S4), to which laminin was not able to bind [21].

Altogether, these data indicate that CNF1 and Laminin do not bind to the same site on Lu/BCAM. Gel filtration and SAXS experiments suggest a 6 to 1 symmetry (Lu/BCAM-CNF1) in the receptor-toxin complex. However, further structural information on the complex is still needed to definitely identify the crucial interaction sites. In humans, Lu plays a role in the vaso-occlusion of red blood cells (RBCs) in sickle cell patients. In sickle RBCs, epinephrine increases the Lu/BCAM-mediated adhesion of erythrocytes to laminin α5 of endothelia [25,26]. Furthermore, Lu/BCAM is highly expressed in epithelial skin tumors [27] as well as in hepatic metastasis from colorectal cancer [28]. Our studies revealed a deeper understanding of the CNF1–Lu/BCAM interaction, which may be used in the development of anti-toxin strategies or therapeutic approaches in the treatment of Lu/BCAM associated diseases.

## 4. Experimental Procedures

### 4.1. Cell Culture

K562 Lu cells were cultivated in RPMI-1640 medium supplemented with 10% fetal calf serum (FCS), 4 mM penicillin, 4 mM streptomycin, and 1 mM Geneticindisulfate (G418).

HeLa cells and COS7 cells were cultivated in Dulbecco’s modified Eagle’s medium (DMEM), supplemented with 10% FCS, 4 mM penicillin, 4 mM streptomycin, as well as 1% non-essential amino acids and 1% sodium pyruvate.

The different cell lines were cultivated at 37 °C with 5% CO_2_ in a humidified atmosphere.

### 4.2. Purification of Recombinant Lu-Fc Proteins

For purification of recombinant Lu-Fc proteins COS7 cells were transfected with a signal pIgplus vector encoding Lu1234-Fc or Lu12345-Fc respectively or a pIgplus MCS vector encoding Lu1-Fc, Lu12-Fc, or Lu123-Fc (provided by Wassim El Nemer, INSERM, Paris, France) by using Lipofectamine 2000 according to the manufacturer’s instructions, using DMEM, with 4 mM penicillin and 4 mM streptomycin. After 4 h of cultivation, the medium was replaced by DMEM with 5% immunoglobulin-depleted fetal calf serum supplemented with 4 mM penicillin, 4 mM streptomycin, 1% non-essential amino acids, and 1% sodium pyruvate. After five days of incubation, the cell culture supernatants containing the Lu-Fc proteins were incubated with Protein A sepharose beads (GE Healthcare, Little Chalfont, UK) at 4 °C overnight. Elution was performed according to the manufacturer’s instructions. To verify the protein purifications, SDS-PAGE following Coomassie staining was performed as well as western blotting using an anti-Fc antibody.

### 4.3. Cloning, Mutagenesis and Purification of Recombinant CNF Proteins

For the generation of GST-CNF1 mutants, quick change PCR was performed by using specific primers and the plasmid pGEX-2TGL+2 encoding GST-CNF1. To produce the CNFY helix mutant (aa 1–719 CNFY, aa 720–733 CNF1, aa 734–1014 CNF1) PCR was used to generate an N-terminal and C-terminal CNFY fragment with overlapping overhangs of around 10 base pairs. In a second PCR one CNFY chimera fragment was generated and was cloned into pGEX-2TGL. The final plasmids were transformed into competent *E. coli* TG1.

To purify recombinant GST fusion proteins, LB-medium supplemented with ampicillin (100 µg/mL) was inoculated with an overnight culture and was cultivated at 37 °C, 180 rpm to an optical density (OD_600_) of around 0.6. To induce protein expression, IPTG was added to a final concentration of 100 µM. The bacteria culture was incubated for additional 12 h at 28 °C, 800 rpm. After collecting the cells by centrifugation for 15 min at 4 °C, 6000× *g* the pellet was solved in lysis buffer (20 mM Tris-HCl, pH 7.3, 10 mM NaCl, 5 mM MgCl_2_, 5 mM DTT, 1 mM PMSF, and 5 µg/mL DNAse) following sonification. After centrifugation for 45 min at 14,000 rpm, the lysate was incubated with Glutathione-Sepharose Beads. Elution was performed according to the manufacturer’s instructions. To verify the protein purification, SDS-PAGE was performed following Coomassie staining.

### 4.4. SDS-PAGE and Western Blotting

For verification of Lu-Fc proteins and CNF purifications, 12.5% SDS-PAGE was performed. In the case of in vitro Rho shifts, 15% urea-SDS-PAGE was used. Either Coomassie staining was done or western blotting by transferring the proteins to a PVDF membrane. Then, the membrane was blocked with 5% skimmed milk. For the detection of the GST-CNF proteins, an anti-GST antibody (1:1000, GE Healthcare, Little Chalfont, UK) for Lu-Fc proteins, an anti-Fc antibody (1:5000, Santa Cruz, CA, USA), and for detection of RhoA an anti-RhoA antibody (1:1500, Cell Signaling, Danvers, MA, USA) was used. With exception of an anti-Fc antibody, a horseradish peroxidase-coupled second antibody for chemiluminescent detection was used.

### 4.5. Dot Blot Binding Studies

For direct interaction studies, dot blot assays were performed by spotting CNF proteins on a nitrocellulose membrane. After incubation with 5 µM rLu/BCAM in TBST (Tris-buffered saline with 0.05% Tween-20), the membrane was blocked with 5% milk. Three washing steps followed before an immunoblot using an anti-Lu/BCAM (1:1000, Abcam, Hong Kong, China) was performed. As a loading control, an anti-GST antibody was used (1:1000, GE Healthcare).

### 4.6. In Vitro Rho Shift

To confirm the catalytic activity of the CNF purifications, a Rho shift analysis was performed. Therefore, the toxin (1 µg) was incubated with 1 µg recombinant RhoA, 1 × CNF1 buffer (10 × CNF1 buffer: 10 mM MgCl_2_, 1 mM DTT, 1 mM EDTA and 50 mM Tris/HCl pH 8) and 1 nM GDP adding demineralized H_2_O up to a total volume of 20 µL. After 4–7 h, samples were taken and an immunoblot was performed by using 12.5% Urea SDS-PAGE and an anti-RhoA antibody. The deamidated RhoA can be detected on an immunoblot by a slight ‘shift’ in comparison to the un-deamidated RhoA.

### 4.7. FACS Analysis

Fluorescent labeling of proteins with DyLight488 Maleimide (Thermo Scientific, Waltham, MA, USA) was performed according to the manufacturer’s instructions. Prior to the labeling, a buffer exchange to PBS with 10% Glycerin pH 7.5 was performed. After that, excess dye was removed using PD-10 Desalting Columns (GE Healthcare).

For analysis of cell surface binding of DyLight488-labeled proteins cells of interest were detached from culture plates with PBS complemented with 10 mM EDTA and were washed twice with PBS. In the case of suspension cells, no detachment was necessary. In most cases, cells were pre-incubated with unlabeled proteins of interest for 30 min at 4 °C in 1 mL DMEM or RPMI-1640 followed by washing for three times with PBS. After this, an incubation with DyLight488-labeled proteins for 30 min at 4 °C in 1 mL DMEM was performed. Following washing for three times with PBS, FACS measurements were performed. Flowing Software (Version 2.5.1, Perttu Terho, Turku Centre for Biotechnology, Turku, Finland, 2013) was used for the analysis of the data.

### 4.8. GST-Based Pulldown Assay

The cell culture supernatants of Lu-Fc expressing COS7 cells were collected, respectively, and were centrifuged for 3 min at 500× *g* to remove dead cells. The supernatants (with equal amounts of Lu proteins) were then incubated with 16 µL of GST-CNF1 coupled Glutathione-Sepharose 4B beads for 25 min at 4 °C. After washing two times with PBS and one time with PBST, beads were incubated with Laemmli sample buffer for 10 min at 95 °C. Finally, the eluted proteins were separated via SDS-PAGE and an immunoblot with an anti-Fc antibody was performed.

### 4.9. Surface Plasmon Resonance Measurements

To investigate dynamic interactions of GST-CNF1 with Lu the Biacore X100 system (Biacore, Little Chalfont, UK) was used. Lu-Fc was amine-coupled to a CM5 sensor chip, according to the manufacturer’s instructions (10 mM sodium acetate pH 4.5). Around 1300 response units were achieved. Sensorgrams were performed using 1 × running buffer (10 × HBS-EP+buffer: 0.1 M HEPES, 1.5 M NaCl, 0.03 M EDTA and 0.5% Surfactant P20), with a flow rate of 30 µL/min at 25 °C. Up to six different concentrations of pure ligand (GST-CNF1) were injected. GST-CNF1 was purified via gel filtration on a Superdex 200 10/300 GL column (GE Healthcare) prior to Biacore measurements. With the help of Biacore X100 Evaluation software (version 2.0) kinetic constants were calculated.

### 4.10. Purification of the Lu/BCAM-CNF1 Complex by Gel Filtration

Recombinant human extracellular rLu/BCAM with Fc-tag was purchased from Hölzel Diagnostika Handels GmbH (Köln, Germany). GST-CNF1 (720–1014) was purified prior to gel filtration via affinity chromatography as explained above. The GST-tag was removed by thrombin cleavage. To purify the complex, CNF1 (720–1014) and rLu/BCAM-Fc were incubated in 50 mM Tris, 150 mM NaCl, 0.01% Tween 80, pH 7.4 at equimolar ratios for 20 min at 4 °C, following centrifugation for 10 min at 20000× *g*. Then, the complex was purified in a final gelfitration step (Äkta Purifier-System, Superdex 200 Increase 10/300 GL column, GE Healthcare, München, Germany). As a protein standard, the gel filtration standard from Biorad (151–1901) was used. For SAXS analysis CNF1 (720–1014) was concentrated to 6 mg/mL, the complex as well as rLu/BCAM-Fc to 2 mg/mL in 50 mM Tris/HCl, 150 mM NaCl, 0.01% Tween 80 (pH 7.4).

### 4.11. Small-Angle X-ray Scattering (SAXS) Analysis of a Lu/BCAM-CNF1 Complex

SAXS measurements were performed on beamline B21 at Diamond Light Source, Didcot, UK. Approximately 50 µL of sample were loaded onto a 4.8 mL KW-403 column (Shodex, Munich, Germany), equilibrated in 20 mM Tris-Cl, 150 mM NaCl, pH 7.6, on an Agilent 1260 system (B21, Diamond Light Source, Didcot, UK). Approximately ~45 μL of sample was injected at 2–6 mg/mL using a flow rate of 160 μL per minute. Samples were CNF1 catalytic unit, rLu/BCAM-Fc, and rLu/BCAM-Fc/CNF1 complex. Chromatographic elution was directed into a specialized SAXS flow cell, with a 1.6 mm path length, held at 20 °C. SAXS measurements were made using a sample to detector distance of 3.9 m and X-ray wavelength of 1 Å. SAXS images (frames) were collected as a continuous set of 3 s exposures across the elution peak and were corrected for variations in beam current, normalized for exposure time and processed into one-dimensional (1D) scattering curves using in-house beamline Software (GDA). The corresponding buffer background measurement for producing the background-subtracted curve was collected at greater than 1.5 column volumes. Buffer subtractions and all other subsequent analysis were performed with the program ScÅtter (http://www.bioisis.net/scatter). The SAXS curves were analzsed with the program DAMMIF from the ATSAS suite [29]. The program DAMMIF generates ab initio bead models by simulated annealing that describe the experimental scattering curves. An average model from the results of seven independent DAMMIF runs was calculated using DAMAVER from the ATSAS suite.

## Supplementary Materials

The following are available online at www.mdpi.com/2072-6651/10/1/3/s1, Figure S1: Aa 720-730 of CNF1 are involved in receptor binding, Figure S2: VCAM does not interact with CNF1, Figure S3: Representative chromatograms of size exclusion chromatography of toxin, receptor and the toxin-receptor complex, Figure S4: CNF1 binds to K562 cells expressing Lu and Lu D343A corresponding to the surface Lu-level.
