# Supplementary material for: High Affinity Binding of Escherichia coli Cytotoxic Necrotizing Factor 1 (CNF1) to Lu/BCAM Adhesion Glycoprotein
Source: Toxins (Basel). 2017 Dec 21;10(1):3. doi: 10.3390/toxins10010003 (PMC5793090; doi:10.3390/toxins10010003)
Supplement: Supplementary file 1 [file toxins-10-00003-s001.pdf]

Supplementary Materials: High Affinity Binding of *Escherichia coli* Cytotoxic Necrotizing Factor 1 (CNF1) to Lu/BCAM Adhesion Glycoprotein

Franziska Reppin, Sylvie Cochet, Wassim El-Nemer, Günter Fritz and Gudula Schmidt

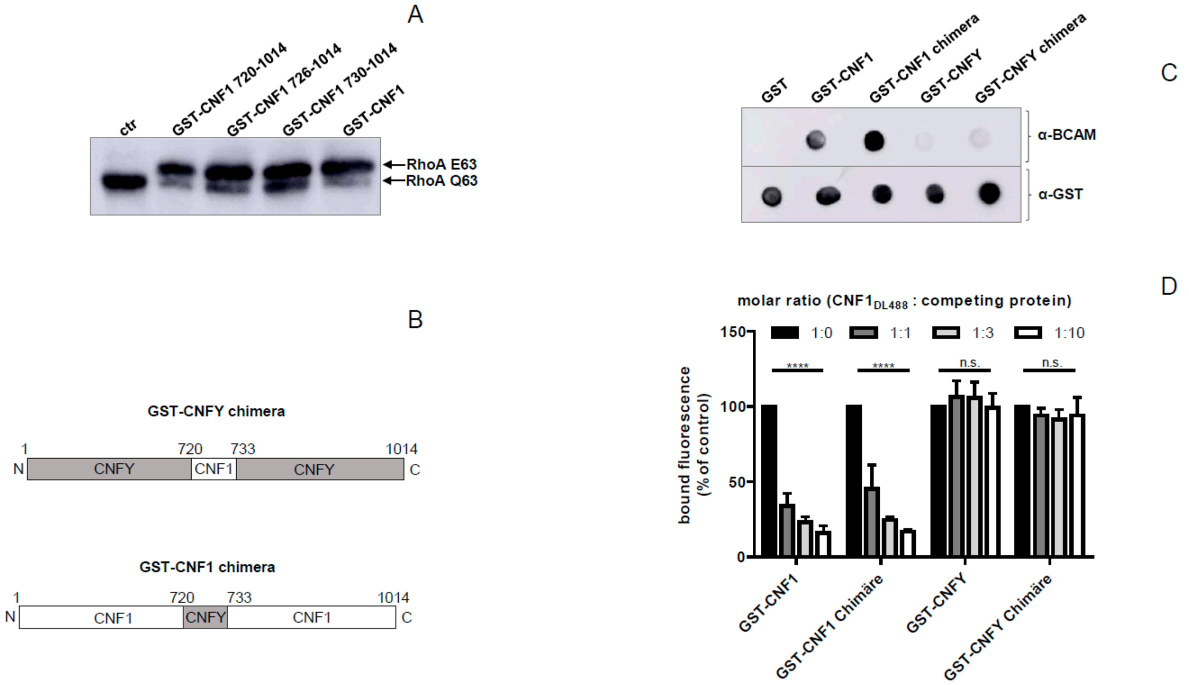

Figure S1. Aa 720-730 of CNF1 are involved in receptor binding. (**A**) in vitro RhoA shift for activity proof. Incubation of GST-CNF1, GST-CNF1 720-1014, GST-CNF1 726-1014 and GST-CNF1 730-1014 with rRhoA followed by SDS-PAGE and immunoblotting using an anti-RhoA antibody. (**B**) schematic representation of the generated GST-CNFY and GST-CNF1 chimera. (**C**) dot blot binding studies, different CNF proteins were dotted on a nitrocellulose membrane and incubated with rLu/BCAM for 30 minutes. After blocking the membrane with skimmed milk, detection of rLu/BCAM was performed by an anti-Lu/BCAM antibody. As a loading control, the membrane was incubated with an anti-GST antibody. (**D**) flow cytometry competition studies, suspension of HeLa cells (3 × 105 cells in 1 mL medium) were preincubated with several unlabeled CNF proteins in different molar ratios respectively. Then, cells were washed with PBS and incubated with DyLight488-labeled GST-CNF1 (as 100 % control cells were incubated with labeled CNF1 without preincubation), Following washing with PBS, cells were subjected to flow cytometry measurements. Bound fluorescence is shown in comparison to the control, which was set to 100 %. Data shown represent three independent experiments + standard deviations. Statistical analyses were performed using two-way ANOVA. ****, $p < 0.0001$.

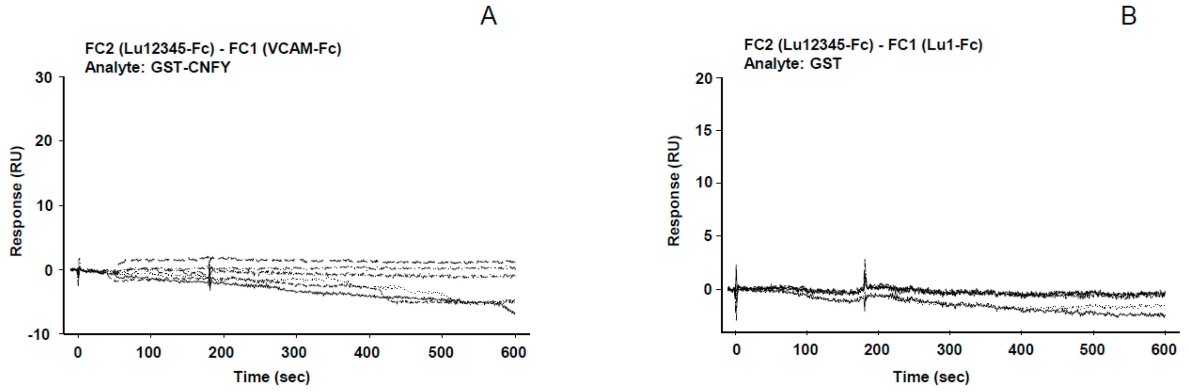

Figure S2. VCAM does not interact with CNF1. (**A**) Binding sensorgram for GST-CNFY interaction with immobilized Lu12345-Fc. VCAM-Fc was immobilized on FC1 on a CM5 sensor chip, Lu12345-Fc on FC2. As a ligand GST-CNFY was injected at 6 different concentrations from 15.6 nM, 31.25 nM, 62.5 nM, 125 nM, 250 nM up to 500 nM. Shown is the sensorgram FC2-FC1. (**B**) Binding sensorgram for GST interaction with immobilized Lu12345-Fc. Lu1-Fc was immobilized on FC1 on a CM5 sensor chip, Lu12345-Fc on FC2. As a ligand GST was injected at 6 different concentrations from 15.6 nM, 31.25 nM, 62.5 nM, 125 nM, 250 nM up to 500 nM.

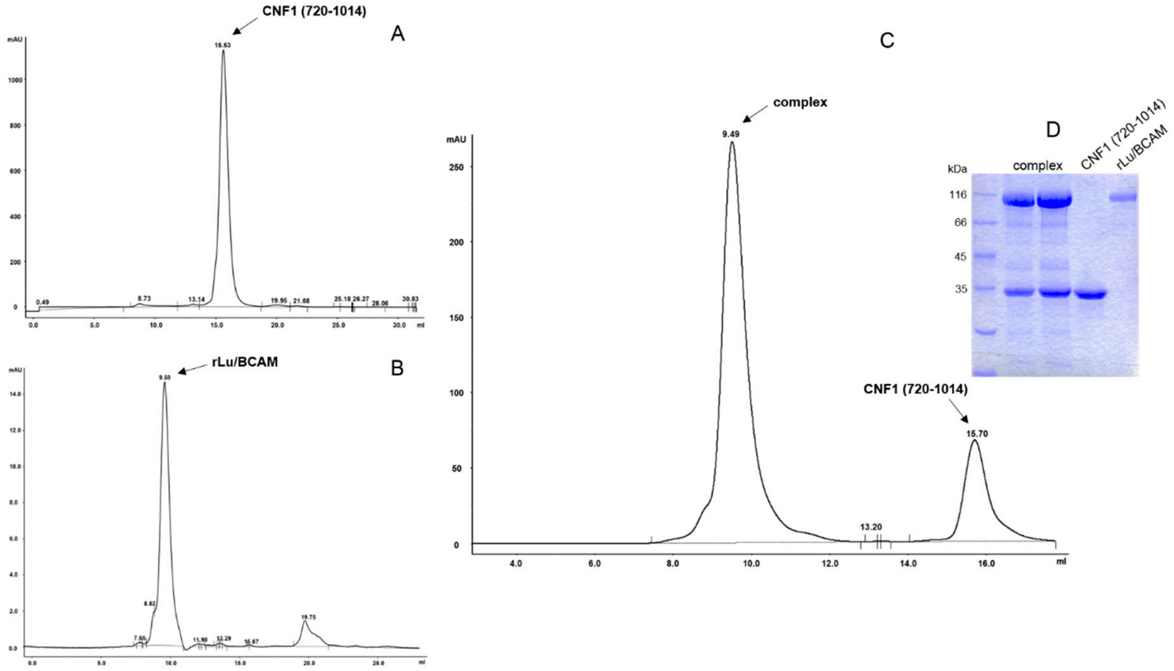

E

	CNF1 (720-1014) [ml]	Complex [ml]
	15.61	9.38
	15.62	9.52
	15.70	9.56
	15.63	9.48
	15.70	9.50
	15.66	9.49
**mean**	**15.65**	**9.49**

Figure S3. Representative chromatograms of size exclusion chromatography of toxin, receptor and the toxin-receptor complex. (**A**) rLu/BCAM-Fc (**B**) and the complex consisting of CNF1 (720-1014) and rLu/BCAM-Fc (**C**) On the x-axis the retention volume is expressed as ml, the y-axis shows the absorbance units (mAU). Peak in graph (**A**): CNF1 (720-1014), Peak in graph B: rLu/BCAM-Fc and peak in graph C: complex CNF1 (720-1014) and rLu/BCAM-Fc. (**D**) different fractions of the size exclusion chromatography verified via SDS-PAGE following Coomassie staining. (**E**) Corresponding retention volume of size exclusion chromatography for each measurement of CNF1 (720-1014) and the complex (rLu/BCAM-Fc with CNF1 (720-1014)) with mean.

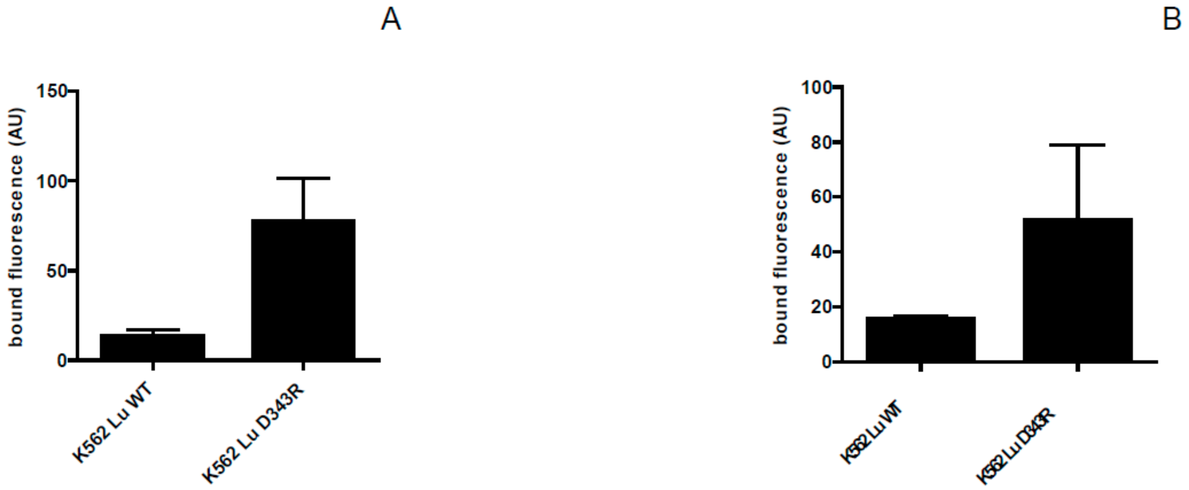

Figure S4. CNF1 binds to K562 cells expressing Lu and Lu D343A corresponding to the surface Lu-level. (**A**) Binding of DyLight488-labeled CNF1 to K562 Lu cells and K562 Lu D343A cells shown in bound fluorescence. Cells were incubated with 2 µg GST-CNF1DL488. After washing with PBS, cells were subjected to flow cytometry measurements. (**B**) surface Lu/BCAM level on K526 Lu and K562 Lu D343A cells respectively. Cells were incubated with an anti-Lu/BCAM antibody and washed with PBS. Following incubation with a DyLight488-labeled secondary antibody, cells were washed with PBS and flow cytometry measurements were performed. Data represent the mean bound fluorescence of three independent experiments + standard deviations.
